# Progress in Novel Electrodeposited Bond Coats for Thermal Barrier Coating Systems

**DOI:** 10.3390/ma14154214

**Published:** 2021-07-28

**Authors:** Kranthi Kumar Maniam, Shiladitya Paul

**Affiliations:** 1 Materials Innovation Centre, School of Engineering, University of Leicester, Leicester LE1 7RH, UK; km508@leicester.ac.uk; 2 Materials and Structural Integrity Technology Group, TWI, Cambridge CB21 6AL, UK

**Keywords:** thermal barrier coatings, gas turbines, aluminium, electrodeposition, aluminides, bond coats, ionic liquids

## Abstract

The increased demand for high performance gas turbine engines has resulted in a continuous search for new base materials and coatings. With the significant developments in nickel-based superalloys, the quest for developments related to thermal barrier coating (TBC) systems is increasing rapidly and is considered a key area of research. Of key importance are the processing routes that can provide the required coating properties when applied on engine components with complex shapes, such as turbine vanes, blades, etc. Despite significant research and development in the coating systems, the scope of electrodeposition as a potential alternative to the conventional methods of producing bond coats has only been realised to a limited extent. Additionally, their effectiveness in prolonging the alloys’ lifetime is not well understood. This review summarises the work on electrodeposition as a coating development method for application in high temperature alloys for gas turbine engines and discusses the progress in the coatings that combine electrodeposition and other processes to achieve desired bond coats. The overall aim of this review is to emphasise the role of electrodeposition as a potential cost-effective alternative to produce bond coats. Besides, the developments in the electrodeposition of aluminium from ionic liquids for potential applications in gas turbines and the nuclear sector, as well as cost considerations and future challenges, are reviewed with the crucial raw materials’ current and future savings scenarios in mind.

## 1. Introduction

High-performance gas turbines have been widely employed as propulsion systems for aeroplanes and large ships, and in power generation. Since the invention of the gas turbine engine, there has been a constant effort to increase its productivity and power. As a result, components for these engines require materials with enhanced mechanical properties at extremely high temperatures [1,2]. Nickel-based superalloys have been the material of choice for high-temperature turbine blades, nozzle guide vanes, and other components. The demand for highly efficient gas turbines in modern aircraft and power generation is driving the engine inlet temperatures to unprecedented levels [3]. As a consequence, the performance of classic turbine engine components, notably turbine blades and rotors operating at higher pressures and temperatures, could no longer match the new requirement, creating a “bottleneck” in the process [4]. In addition, hot-end components of gas turbines must withstand extreme conditions, such as high-temperature oxidation, erosion, stresses and corrosion while in service [5].

One of the most serious environmental issues confronting the globe today is an increase in greenhouse gas emissions (especially CO_2_) as a result of the extensive use of fossil fuels. Increasing the gas inlet temperature and operating pressure in turbines and boilers to increase their efficiencies is an approach to reduce the overall CO_2_ emissions in numerous sectors. However, to achieve this, one needs to develop materials capable of long-term performance under these harsh conditions [6]. Nickel-based superalloys of various generations are predominantly used in various components of high-temperature systems due to their mechanical strength and excellent creep resistance. Despite these benefits, these superalloys have inadequate oxidation resistance and suffer from type II hot corrosion (850–1100 °C) [7,8].

**Figure 1:**
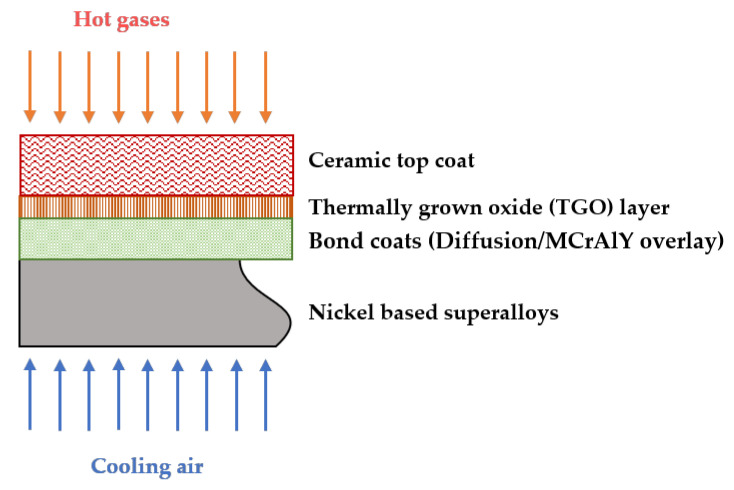
Figure showing the model representation of a TBC system.

The introduction of next-generation jet engines with better thrust-to-weight ratios has been a pivotal point in the aviation industry. These engines operate under higher gas inlet temperatures in the combustion chambers and turbines. To protect the surfaces of metallic parts operating at such high temperatures, thermal barrier coatings (TBCs) have become the preferred industry choice and have been applied in gas turbines, aircraft propulsion, marine and so on [9,10]. Market forecasts predict a notable production of ~228,000 gas turbine engines for aviation purposes and ~5480 gas turbine engines for power generation with a total projected value of USD 1.3 trillion by 2030 [11,12]. Considering these data, it is reasonable to foresee an increase in demand for TBC systems in the near future. A typical TBC system consists of four layers: (i) metallic substrate at the bottom (generally Ni-superalloy), (ii) an intermediate bond coat (BC), (iii) thermally grown (Al_2_O_3_) oxide (TGO) formed during service, and (iv) a ceramic top coat [12,13]. A schematic TBC system model is represented in Figure 1. A TBC system is complex in nature and combines metallic and ceramic components together to perform under extremely challenging thermal loading conditions. The top coat is the outermost layer, generally made of yttria partially stabilised zirconia (YPSZ) containing ~7 wt % yttria with a thermal expansion of 9–10 mm·m^−1^ [2,14]. The ceramic top coat, which is directly in contact with the aggressive environment, prevents degradation and also protects the underlying layers during thermal cycling of engine components [9,15].

The metallic bond coat (BC) is an oxidation resistant intermediate layer between the ceramic top coat and the metal-alloy substrate, whose primary role is to protect the superalloy substrate from oxidation and hot corrosion. Besides possessing a thermal expansion of 13–14 mm·m^−1^ at temperatures in the range of 1100–1300 K [16], it also plays a critical role in balancing thermal mismatch between the base metal-alloy substrate (13–13.5 mm·m^−1^) and top coat (9–10 mm·m^−1^) [14]. This thermal mismatch is a result of the difference in coefficients of thermal expansion [17,18]. The bond coat is arguably the most crucial component of TBCs as the coating system performance is usually linked to the bond coat creep and yield properties governed by its composition and microstructure, thereby dictating the failure behaviour of TBCs [19]. Although the bond coat is mostly metallic, the presence of intermetallic phases plays a role in its performance. The two most commonly used high-temperature coatings for protection against oxidation and hot corrosion are diffusion aluminide and MCrAlY coatings (M = Co, Ni or Co + Ni). Formation of intermetallic coatings may be achieved by a combination of processes such as physical and chemical vapour deposition (CVD), pack cementation, spraying (low pressure plasma, high velocity oxy-fuel, high velocity air fuel), and electroplating followed by subsequent heat treatments [20,21,22]. Among these, electroplating is realised as an effective “non-line-of-sight” coating process strategy to achieve the diffusion aluminide coatings [23] and overlay coatings [24], owing to its simplicity of operation, high purity deposit formation, significantly low cost and capability of coating complex objects homogeneously, even on the inner surface of tubes [25]. Electrodeposition of metallic coatings from ionic liquids (ILs) was demonstrated to be feasible, and as a result ILs offer an alternative electrolyte media and are a topic of interest for many industries [26,27,28,29,30,31].

The aim of this paper is to provide a review of progress in the development of bond coats using ionic liquids (ILs) for thermal barrier and tritium permeation barrier coatings. The review is divided into four parts (Section 2, Section 3, Section 4 and Section 5 of the paper). Section 2 provides an overview on the works that utilised electrodeposition/electroplating to develop the bond coats (diffusion, overlay coats) from aqueous medium for thermal barrier coating applications. Section 3 introduces the concept of ionic liquids and their progress in potential application as an electrolyte media to develop bond (diffusion aluminide) coats for: (i) thermal barrier coatings, and (ii) tritium permeation barrier coatings. A brief summary of the tritium permeation barrier coatings will also be discussed in Section 3. Section 4 provides the cost considerations and future challenges associated with the development of bond coats using ILs. The final section (Section 5) will summarise the findings and provide concluding remarks, highlighting the future roadmap.

## 2. The Role of Electrodeposition in Thermal Barrier Coatings

To improve the resistance against high temperature oxidation and corrosion, intermediate metallic bond coats, such as diffusion aluminides or MCrAlY overlays, are applied over the critical Ni-based superalloy components [32]. The bond coats are deposited on Ni-based superalloys, and these form either chromia or alumina due to oxidation in service. The bond coats can be broadly classified based on the major phase constituent: (i) β-NiAl, (ii) fcc γ-Ni, (iii) γ-Al, (iv) γ′-Ni_3_Al, and (v) α-Cr. Coatings consisting primarily of the β-NiAl phase are typically referred to as nickel aluminide coatings and possess an ordered body centered cubic (BCC) structure. During high temperature exposure, they also serve as a source of Al for the development and maintenance of the protective alumina (Al_2_O_3_) scale on the surface [17,33,34,35].

### 2.1. Diffusion Aluminides

Diffusion aluminide coatings are generally formed by the interdiffusion of alloying elements from the superalloys and an aluminium source (usually an Al donor) which protects the substrate superalloy from high temperature oxidation. Diffusion aluminide bond coatings are typically formed through incorporation of Al onto the components’ surface by employing a combination of processes (mentioned in earlier sections) involving diffusion of Al [36], with pack cementation being predominantly studied [2,21,37]. However, oxidation resistance of the coating has been shown to be affected by oxide scale spallation due to an accelerated depletion of Al from the coating during thermal cycling. To improve the scale adhesion, aluminide coatings have been modified with elements such as Ce, Cr, Hf, Pd, Pt, Y, Zr [8,23,38,39,40,41,42,43,44,45]. It has been identified that Pt-modified aluminide coatings are effective and provide excellent oxidation resistance at high temperatures relative to their un-modified counterparts [34,40,41]. Therefore, these coatings have been the focus of extensive research during the past several decades and numerous aspects of Pt-modified aluminide coatings have been exhaustively investigated. Amongst them, incorporating an electrodeposited intermediate layer followed by aluminising treatment (CVD) was identified as one of the beneficial strategies owing to its low cost, simplicity of operation and capability of coating complex shaped parts of an industrial gas turbine [21,37,46].

Research attempts were also made by performing electrodeposition of different combinations of metals followed by addition of rare earths (such as Zr [44,47], Hf [4,48], and Ce; and their oxides) [49]. The incorporation of all the above-mentioned elements (Pt, Pd, Zr, Hf) and their combination through electroplating followed by diffusion aluminising was demonstrated to influence the oxidation resistance of aluminide coatings positively. However, the mechanisms of protection and their roles differ significantly. Replacement of some of the above with cost-effective alternatives, such as Pd, was shown to play a critical role in eliminating the rumpling tendency when combined with Pt. Several aluminide coatings fabricated by aluminising the electroplated metals (Pt, Pd, Pt + Pd) previously deposited on different superalloys (with or without incorporating dopants such as Zr, Hf, Y) have been shown to contribute to the following besides improving oxidation resistance and reducing rumpling tendency of the oxide (Al_2_O_3_) scale [50].

- Retards the phase transformation from θ-phase (Al_2_O_3_) to α-phase (Al_2_O_3_).
- Delays phase transformation and degradation rate from β-NiAl phase to γ′-NiAl phase.
- Increases the Young’s modulus of Pt-modified β-NiAl coating.
- Good abilities to fix/stabilise sulphur (S), and prevents ‘S’ segregation and extinct Cl during its exposure to different corrosive environments (such as sulfate salt, chloride salt).

In addition to the above-mentioned metals and their combinations to produce intermediate electrodeposited layers, attempts were also made to create electrodeposited Ni and Ni-Co-modified coatings to function as diffusion aluminide coatings on post-treatment via the cementation, CVD techniques and so on. For instance, Zakeri [36] reported the preparation of Ni-Co-modified aluminide coatings on the Hastealloy-X substrate by a combined process of electrodeposition and slurry aluminizing. In this regard, pure layers of Ni and Ni-50 wt %Co were initially applied via an electrodeposition process and successive aluminising was carried out by a slurry technique. The results of these analyses revealed that a compact and dense aluminide coating was formed with a two-layered structure containing the outer Al-rich β phase and inner interdiffusion zone. While the primary electrodeposited Ni layer served to improve the oxidation resistance and limit the formation of Kirkendall pores, alloying with 50 wt % Co (to form electrodeposited Ni-Co) enhanced the hot corrosion performance when exposed to sulfate-rich environments by preventing the internal sulfidation. Realising the benefits of pre-deposited Ni layer, the research works were further extended on ferritic-martensitic steels to form Ni-Al and Fe-Al coatings. The performance in terms of oxidation resistance and hot corrosion resistance in a marine environment was also investigated [51]. It was observed that Ni-based diffusion aluminide coatings exhibited better corrosion resistance than Fe-based ones [51]. The superior performance was attributed to the beneficial effects of the Ni-plating layer which played an inhibitory role towards self-sustaining oxy-chlorination, tungsten (present in base metal substrate) outward diffusion and interdiffusion behaviour.

### 2.2. Overlay Coatings

Though diffusion aluminide coatings were extensively studied using an intermediate electroplated layer (Pt, Pd, Pt + Pd) with or without reactive element (Zr, Hf) doping, they still suffer from certain drawbacks, such as rumpling/wrinkling tendency of the grown oxide layers, spallation of ceramic top coat, prone to cracking and so on [4,8,34,35,44,47,50]. Subsequently, focus has been shifted to the development of MCrAlY overlay coatings (M = Ni, Co, or Ni + Co) as they offer superior mechanical properties, flexibility in terms of composition selection, and facilitate creation of coatings with balanced combinations of coating properties. The lower ductile-to-brittle transition temperature (DBTT) of the MCrAlY overlay coatings combined with the high chromium content make them resist to cracks (during thermal cycling) and corrosion (under hot conditions). However, MCrAlY overlay coatings have a tendency to exhibit poor oxidation resistance due to the lower aluminium content relative to aluminide coatings. As a consequence, these coatings would experience a deterioration in their mechanical properties due to the outward diffusion of certain elements such as W and Mo from the substrate, thereby drastically increasing the brittleness [7].

To improve the oxidation and corrosion resistance properties of these overlay coatings under high temperature conditions, studies that were conducted included either modifying the MCrAlY coatings with Pt [32], or introducing an intermediate diffusion barrier, such as electroplated Re-Ni, between the MCrAlY and the superalloy substrate. On the one hand, results from Pt-modified MCrAlY coatings showed improved oxidation and corrosion resistance, while on the other hand, the presence of an intermediate electrodeposited Re-Ni barrier was shown to influence the mechanical properties (adhesion strength) and thermal shock resistance. Additionally, the authors observed a reduction in the mismatch of the thermal coefficient of expansion between the intermediate electrodeposited Re-Ni barrier and the MCrAlY overlay coating. The electrodeposited Re-Ni reduced the substrate surface roughness which contributed to the improved adhesion strength of the coatings [52]. Aliabadi et al. [6] proposed a novel method of producing a high-temperature NiCoCrAlY overlay coating system whose desired properties could be achieved gradually after the application of aluminide coatings on NiCoCrAlY overlay coatings; they referred to them as graded aluminised NiCoCrAlY overlay coatings. These were prepared by introducing the NiCoCrAlY coatings first by state-of-the-art techniques such as thermal spray process followed by electrodeposition of Ni-CeO_2_ layer which was then subjected to aluminisation via low pressure CVD. The authors successfully achieved the desired β-NiAl phase which was reported to be uniform throughout. On comparing the mechanisms of the coatings with and without the electrodeposited Ni-CeO_2_ layer, the growth mechanism in the presence of an electrodeposited Ni-CeO_2_ layer was reported to be significantly different. The aluminide growth in the presence of an electrodeposited Ni-CeO_2_ layer was shown to be primarily governed by the diffusion of Al inward followed by the outward diffusion of Ni, highlighting the beneficial influence of the electrodeposited Ni-CeO_2_ layer.

Yang et al. [53,54] investigated the role of electrodeposited Pt in Pt-modified NiCoCrAlY coatings in improving the oxidation resistance. To understand the influence, Pt-modified NiCoCrAlY coatings were prepared by two different approaches: (i) deposition of NiCoCrAlY coating by arc ion plating on previously electrodeposited Pt layer (NiCoCrAlY + Pt), and (ii) electrodeposition of Pt layer on previously deposited NiCoCrAlY coating by arc ion plating (Pt + NiCoCrAlY). The Pt-modified NiCoCrAlY coating’s synthesis via the later approach (Pt + NiCoCrAlY) was shown to favour the formation of α-Al_2_O_3_ with reduced TGO growth rate, and the results displayed an overall improvement in overall oxidation performance. The study also confirmed the extended service life of 4 mol% YSZ TBCs and, the impurity phases α-Cr and δ-(Cr,Co,Ni) were eliminated. The study demonstrated significant effort towards optimising the location of the Pt-rich region in the Pt-modified overlay coating. Because of the various benefits associated with the addition of Pt to the TBC system, identifying a suitable Pt-rich location is critical for maximising the benefits of electrodeposited Pt.

**Figure 2:**
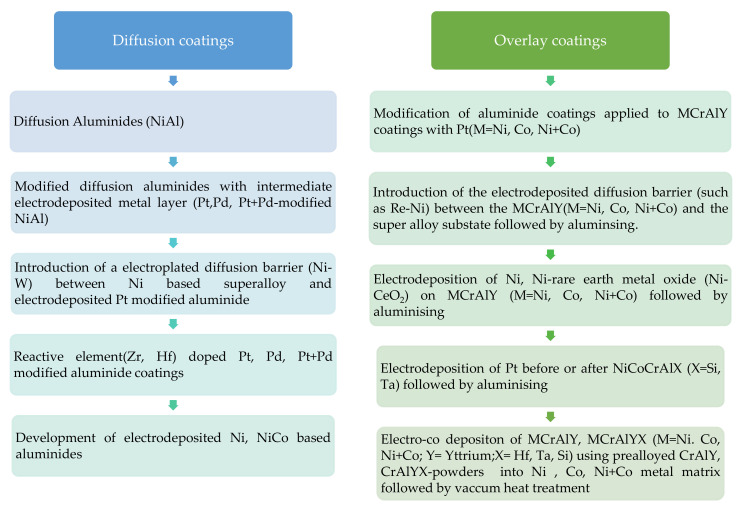
The evolution of bond coats utilising electrodeposition method as an effective strategy.

Different from the state-of-the-art techniques and the above-mentioned electrodeposition methods, Zhang’s group [19,24,55] reported the fabrication of MCrAlY and MCrAlYTa (M = Ni, Co, Ni + Co) coatings on ReNe80 and CMSX-4-based superalloys via electrolytic co-deposition, which was claimed to be one of the promising alternatives. Two types of prealloyed CrAlY and CrAlYTa powders prepared by atomization and ball milling were introduced into a plating bath containing Ni-salts and Co-salts. Electro co-deposition was then performed employing the in-house designed patented barrel plating. Diffusion heat treatment at high temperatures was subsequently conducted to obtain the MCrAlY coatings with a desired microstructure consisting of multiple phases [19,24,55]: (i) β-(Ni,Co)Al and (ii) γ-Ni(Co). Besides being low cost and a non-line-of-sight process, the methodology adopted in this study was demonstrated to be techno-commercially competitive to electron beam-physical vapor deposition (EB-PVD) and thermal spray processes. High-temperature coatings produced via the two-step electro-co-deposition and diffusion heat-treatment exhibited superior oxidation resistance and hot corrosion resistance (Type I: 650–800 °C). Besides, the authors also confirmed that there was no formation of precipitates containing refractory metals. A summary of the evolution of electrodeposition of metals that have been utilised to develop bond coats for thermal barrier coating applications is shown in Figure 2. Table 1 lists the recent works on bond coats that were prepared employing electrodeposition.

## 3. Ionic Liquid Assisted Electrodeposition—A Way Forward

Diffusion coatings have been widely applied on complex-shaped parts such as gas turbine blades, vanes, etc., due to their numerous advantages in terms of cost, ease of operation, and utilisation of established techniques, with pack cementation being the most widely used methodology in industries. One of the effective ways of achieving aluminide (Ni-Al) diffusion coatings is through diffusion of pure aluminium layers into the Ni-based superalloys, which requires the deposition of pure aluminium layers. Such a design offers multiple benefits over other methods such as: high purity coatings with good deposition rates, simple operation with low consumption of energy [27,59]. For these reasons, the industrial development of the Al-plating process is growing rapidly and is foreseen as an interesting technology for aerospace and automotive applications [60,61,62]. To create highly pure Al-rich coatings, electrodeposition of aluminium employing ionic liquids as an electrolyte medium can be considered as the preferred choice due to its exceptional properties, such as non-volatility and non-flammability, wide electrochemical potential window, high ionic conductivity, and low vapour pressure. ILs are generally formed from large sized cations and small sized inorganic anions and are asymmetric in nature, and can behave as liquids at room temperature. Earlier attempts to produce Ni-based aluminides from an imidazolium-based IL: 1-methyl-3 ethyl imidazolium chloride (MEImCl) was reported by Pitner et al. [63] and the results demonstrated its potential for application in aerospace. The same group was successful in achieving nickel aluminide coatings with different aluminium contents from MEImCl-based electrolyte containing AlCl_3_ and NiCl_2_. Subsequently, research was conducted to produce aluminide coatings utilising the Co-Al [64,65,66], Cr-Al [42,67] alloys electrodeposited from a variety of ionic liquids: MEImCl, 1-ethyl-3-methyl-imidazolium chloride EMImCl, n-butyl pyridinium chloride BPyCl, trimethylphenylammonium chloride TMPAC. Although the process of Al electrodeposition from ILs requires moisture-free environments, the possibility of achieving high purity coatings with relatively cheap instrumentation and excellent deposition rates over the available vacuum-based techniques makes it unique.

Though there are numerous reports that highlight the electrodeposition of Al from a variety of ILs, the utilisation of Al electroplating from ionic liquids as an electrolyte to produce diffusion aluminide coatings on nickel-based superalloys as an alternative to conventional CVD methods remains a challenge. University of Cranfield [62] was the first group who demonstrated the development of aluminide coatings on Ni-superalloys, combining the electrodeposition of Al from ionic liquids and subsequent heat treatment to form nickel aluminide bond coats which could possibly be overcoated with ceramic top coats for their application as a TBC system in turbine engines. As a part of the EU FP6 project IOLISURF, it was shown that it is possible to achieve β-NiAl diffusion aluminide coatings by heat treating the electrodeposited Al from imidazolium-based IL: EMImCl with a new novel low temperature regime (developed during the project), which involves the interaction of diffusion processes to form the desired diffusion aluminide. The results from the IOLISURF [61] project demonstrated the feasibility of obtaining electrodeposited aluminium from ionic liquids in a production environment. This also indicated the techno-commercial capability of the process to electroplate turbine blades and large components to produce diffusion aluminide bond coats for TBC systems. Realizing the potential benefits of developing aluminide coatings employing the electrodeposited Al from ILs, studies were extended towards utilizing the technology for other energy generation applications, such as tritium permeation barrier (TPB) coatings in nuclear fusion reactors. Because of their reduced thermal expansion mismatch, aluminide coatings with Al_2_O_3_ on the surface of structural materials were chosen as the most viable TPB coating system on a variety of substrates ranging from steels to vanadium alloys. For instance, Xue et al. [25] prepared aluminide coatings on 316 L stainless steel tube internal surfaces employing imidazolium-based IL: EMImCl-AlCl_3_ for the deposition of Al. It was shown that the thickness of aluminide coatings could be potentially varied by regulating the deposition thickness of Al during electrodeposition. Additionally, the ultrasonication results of the electrodeposited aluminium indicated that Al coating and substrate had good adhesion.

Peng et al. [68] reported on the preparation of Al_x_V_y_ aluminide coatings, on vanadium alloy (V-5%Cr-5%Ti), utilizing the two-step aluminizing process which involves the electrodeposition of Al from EMImCl-AlCl_3_ followed by subsequent heat treatment. Such an aluminide formation was shown to be the critical step for the formation of V-Al/Al_2_O_3_ as TPB on V alloy. Zhang et al. [69] further proved that it could be possible to achieve the formation of aluminide coatings on the inner walls of the pipeline through Al electrodeposition from IL-based systems, and that they display excellent tritium resistance performance. However, it was observed that various factors such as: (i) pretreatment type, (ii) aluminium coating thickness, and (iii) metal-alloy substrate, influenced the microstructure and performance of the coatings. These suggest that it is important to control the electrodeposition parameters as they play a key role in achieving the ideal aluminide coatings for intended applications and enhanced performance. Caporali’s group [60] designed a novel aluminising process utilising room temperature Al-electrodeposition from imidazolium-based IL: 1-butyl-3-methyl-imidazolium chloride BMImCl-AlCl_3_ and vacuum diffusion heat treatment at 1000 °C. The aluminising process methodology was applied on bare and CoNiCrAlY-coated Inconel 738 Ni alloy (IN738). The work was a part of the EU FP7 funded project “SCAIL-UP” [70], which proposed a two-step aluminising process based on (a) the aluminium electrodeposition from ILs and (b) subsequent heat treatment to obtain Ni–Al intermetallics. Two samples with the following configurations were studied:

1. Route 1: IN 738 + 10 µm Al deposited from IL + post-treatment (diffusion heat treatment at 1000 °C).
2. Route 2: IN 738 + CoCrAlY coating + 20 µm Al deposited from IL + post-treatment (diffusion heat treatment at 1000 °C).

It was demonstrated that aluminide coatings obtained by electrodeposition of Al on CoCrAlY via route 2 displayed comparable oxidation resistance relative to the aluminides obtained by pack cementation. In contrast, aluminides obtained by the electrodeposition of Al on bare IN738 superalloy through route 1 exhibited poor oxidation resistance relative to the pack cementation-based aluminides. The results from this work as a part of the project signify the benefits of a two-step aluminizing process obtained via the electrodeposition of Al from ionic liquids. Such an approach is beneficial to produce a coating that can offer a simple operation relative to spray techniques and offers the following advantages when aluminides are produced via the above route (route 2):

1. Enhance the oxidation resistance at high temperatures.
2. Facilitates better diffusion of the Al toward the coating and eliminates the formation of carbides, free from W, Ta, Ti that arise from the substrate superalloys.
3. Reduces the high Ni interdiffusion from IN738 to the coating.

**Figure 3:**
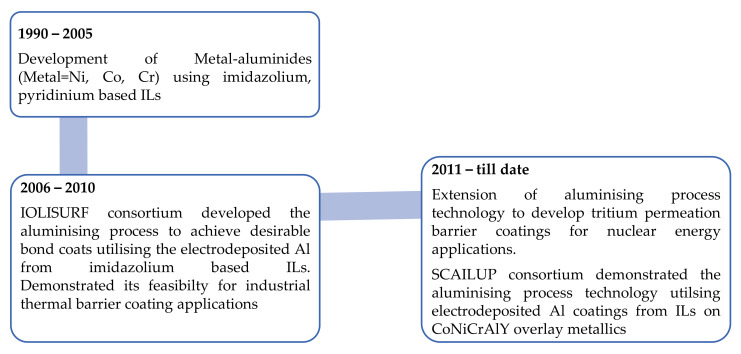
The schematic of the developments associated with aluminide coatings utilising the electrodeposited Al from ionic liquids.

Overall, the aluminising process developed by electrodepositing Al from ILs can be considered as a feasible and suitable technology to produce diffusion aluminides (β-NiAl, Al_x_V_y_) or MCrAlY (M = Ni, Co, Ni + Co) metallic coatings as potential desirable bond coats for high temperature oxidation protection. However, the suitability and adaptability for coating more complex shaped structural parts and the performance of the inner side coatings (inner side of the vanes, pipelines) needs further study. Figure 3 shows the progress in the development of aluminide coatings utilising the electrodeposited Al from ionic liquids.

## 4. Cost Considerations and Future Challenges

**Figure 4:**
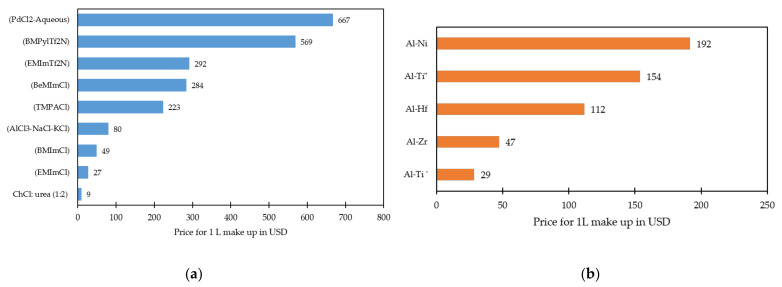
Plots showing the cost comparison of the (**a**) ionic liquid-based systems, an inorganic molten salt system for the electrodeposition of Al [31,72,73,74,75], and aqueous electrolyte for the electrodeposition of Pd [48]; (**b**) the ionic liquid-based systems for the electrodeposition of Al-alloys [71], considering the initial make up of 1 L solution. Electrodeposition of Al-Ti alloy from solution containing TiCl_2_ and TiCl_4_ is represented as Al-Ti’ and Al-Ti”, respectively. Price listings for the ionic liquid electrolytes refer to the time period from January 2021 till date.

In general, over the past decade aluminide coatings prepared by IL-based electroplating technology have been demonstrated to be a potential technology. As a result, the technology is attracting significant interest in enhancing the performance of thermal barrier coating systems and tritium permeation barrier coatings. Such a technique could be deemed advantageous for retaining the mechanical integrity of coated metal-superalloy components, particularly thin-walled turbine components. Considering the potential benefits of electroplating of Al from ILs, it becomes important to understand the costs associated with the preparation of IL-based electrolytes and deep eutectic solvents (DESs) that are used for the electrodeposition of Al and Al-alloys. DESs are a class of ILs (usually low melting point eutectic mixtures) that are formed by mixing hydrogen bond acceptors and hydrogen bond donors. Such systems have been used in electroplating [71]. Figure 4a compares the cost of preparing a 1 L plating solution using ILs that are employed for the electrodeposition of Al from the commercially developed ILs and the ones that were demonstrated to be potentially viable. The cost required for the makeup is calculated and presented considering the volume of individual constituents that are required to be added to prepare 1 L fresh IL solution by considering the IL systems from the references [31,72,73,74], and the value of each constituent is calculated based on the known market price. From the Figure 4a, it can be noted that the make-up cost for ChCl:urea-based IL is the least among the ILs. This demonstrates the economic competitiveness of choline-based ILs besides its technical advantages. To justify the economic feasibility of IL systems at a practical scale, the cost of the AlCl_3_-NaCl-KCl inorganic molten salt system [75] and palladium chloride-based electrolyte [48] is also calculated and presented in Figure 4. Since the BPyrCl-AlCl_3_-based system and platinum electroplating solutions are too expensive, they are not presented in the figure as this would overtly skew the data.

It is understood from Figure 4a that the costs of certain imidazolium-based ILs such as EMImCl/BMImCl are either comparable or lower than the molten salt electrolyte media, justifying the techno-commercial competitiveness of the ILs. Though the cost calculated for other ILs seems uneconomical, increasing the production scale combined with their judicious selection could possibly help to achieve the average IL cost in the order of a few US dollars [76]. This will enable them to compete economically with conventional solvent electrolyte media. Reusing the major fraction of previous-batch plating solution followed by the optimum addition of fresh chemicals and metal precursors/salts could also reduce the recycling costs. Additionally, this process could also replace the expensive platinum group metals (such as Pt, Pd) and contribute to the cost reduction strategy while improving the properties required for their intended use in TBC systems. However, deposition of Al in ILs requires a set up that includes materials that can withstand the corrosive IL environment, inert atmosphere, and harsh operating conditions. For instance, the tank material and mild steel agitator corrode easily in ILs and might need to be replaced with poly (vinyl chloride)/polypropylene tanks and polymeric agitators with appropriate thickness. This might add initial expenditure for setting up IL-based plating lines and would lead to excessive maintenance and repair costs besides frequent operations break-down. Fortunately, the majority of the plating plants constructed so far for the deposition of Al in ionic liquids employed polymeric materials, such as polyethylene, polypropylene, polyvinylchloride, and so on. Another limitation to consider is that pumping an IL solution may be a limiting factor in the industrial application of ILs owing to their highly viscous nature and might incur additional pumping and labour costs [59,77,78]. Performing Al deposition in such an environment might incur problems such as high energy consumption, environmental pollution, and lower energy efficiency. With the increasing demand for and interest in ILs, only a few companies including Iolitec and BASF can deliver ILs on the bulk scale with competitive pricing. The major hindrance to commercialisation of the Al electroplating process from ILs is their reaction on exposure to atmosphere. Employing air- and water-stable ionic liquids for aluminium electrodeposition could be expected to contribute to rapid industrialisation, facilitate coating of larger parts more easily, increase throughput and reduce the overall cost. As can be seen, the costs of recently developed ILs and DESs are comparatively cheaper, indicating the prominence of ILs and their associated benefits with Al electroplating technology. The cost considerations and the earlier studies demonstrate the feasibility of achieving desirable bond coats through electrodeposition of aluminium on different substrates (Ni-alloys, Ti-alloys) from ionic liquids within a production environment to form the respective diffusion aluminides. To provide a better insight, it is worth evaluating the costs that might be incurred to prepare the initial make-up of 1 L ionic liquid electrolytes for the electrodeposition of Al-alloys (Figure 4b). As can be noticed from Figure 4b, replacement of aqueous electrodeposition involving platinum group metals with Al electrodeposition from ionic liquids would be a beneficial option for potential bond coating technology.

Despite the exhaustive development of the electrodeposition of Al-alloys such as Al-Ti [79,80,81], Al-Ni [82], Al-Hf [83], and Al-Zr [84] from ionic liquids, the possible formation of diffusion aluminides, their resistance against oxidation, hot corrosion and possible applications in TBC systems are yet to be studied. Moreover, the ability to electroplate turbine blades and large components with Al and Al-alloys employing economic IL-based systems requires proper control of the structure to improve the performance of the coatings produced through this technology. Therefore, understanding the structure–property relation of the electrodeposited aluminium from the existing, newly developed ILs and the approaches to control the process parameters to achieve the aluminide coatings for their possible application in thermal barrier and tritium permeation barrier coatings necessitates further research [1].

Many of the works reported on in this review contain critical raw materials (CRM) either in the coatings (Pt, Pd) or in the base alloys (Re, Ru, Cr etc in Ni super alloys, Ti alloys). It is also recognised that because of the huge number of critical or semi-critical material components present in gas turbines, as well as their economic relevance for EU markets, numerous scenarios should be developed to achieve reasonable CRM savings [2,85]. Considering the least amount of supply risk associated with aluminium, developing Al electroplating from ionic liquids could be considered as a beneficial approach to produce diffusion aluminides which serve as bond coats to enhance the service life of the newly developed alloys as base substrates. However, their proper optimization associated with the continuous recent advances in IL-based electroplating technologies needs a systematic study. Adopting the IL-based Al electroplating technology is expected to contribute to enhancing the durable life of the coatings applied on the underlying metal-alloy substrates and CRM savings while minimising the environmental impact. However, to achieve these, it is important to integrate different aspects of the electroplating technology, such as (i) optimization of suitable IL systems, (ii) process methodology, (iii) surface pretreatments, (iv) development of Al and Al-alloy electroplated coatings with optimum compositions. However, the question on how to integrate them will be a key challenge to be addressed.

## 5. Conclusions and Outlook

This review provides a survey of the bond coatings involving electrodeposition of metals from aqueous media and ionic liquids for their possible application as barrier coatings in gas turbines and other power generation systems. The review emphasises the importance of utilising electrodeposition for producing bond coats (diffusion and overlay), and their developments utilising both the systems (aqueous and ILs) are discussed in detail. Electrodeposition could certainly be considered as a promising, low-cost and “non-line-of sight” coating process which has the ability to produce desirable bond coats with good deposition rate and enhanced performance. Works based on the modification of the aluminides with intermediate electrodeposited layer with appropriate doping of rare earth metals and oxides demonstrate an increase in resistance against oxidation and hot corrosion but poses certain limitations. As an alternative strategy, approaches proposed by various researchers which dealt with the electrodeposition of Pt on MCrAlZ (M = Ni, Co, Ni +Co; Z = Y, Si, Ta) overlays and vice-versa, electro-co-deposition of MCrAlY and MCrAlYX (M = Ni, Co, Ni + Co; X = Si, Ta, Hf) utilizing prealloyed CrAlY, and CrAlYX- powders and their performance were discussed. The cost benefits associated with IL-assisted aluminium electroplating technology for producing diffusion aluminide coatings were discussed considering the potential for the aluminising process’ development. Developing Al electroplating from ionic liquids could be considered as a beneficial approach to produce diffusion aluminides, which serve as bond coats to enhance the service life of the newly developed alloys as base substrates. Despite the significant developments associated with the electrodeposition of Al-alloys, such as Al-Ni, Al-Hf, Al-Ti, Al-Zr from ILs, optimization of the technology for their possible application in barrier coatings requires further study. With the continuous recent advances in IL-based electroplating technologies, it is important to understand the structure–property relation and its influence on the performance of the real-time Al and Al-alloy electroplated industrial complex parts, such as turbine blades, large components, etc. Novel designing approaches to control these parameters are necessary to achieve ideal aluminide coatings for their possible applications in gas turbines and tritium permeation barrier coatings. Such an approach is expected to contribute to the real-time protection of the underlying bulk alloys, with consequent increased lifetime and operational performance of the coated components. The review also discussed the critical raw material content in substrate alloys and coatings, and suggested possible feasible solutions for CRM savings. However, further research is needed to understand, analyse and determine the suitability of Al electrodeposition to coat complex shaped parts, and the performance of the internal surface coatings (inner side of vanes, tubes) in real-time operations.

## Figures and Tables

**Table 1:** The bond coatings prepared by electrodepositing metals with or without incorporating dopants employing different aluminising treatments.

Type of Bond Coating	Substrate	Diffusion Barrier	Type of Aluminising	Reference
Overlay	Hastealloy X superalloy	NiCoCrAlY with Electroplated Re-Ni	CVD	[52]
Overlay	Inconel 738 LC superalloy	Electroplated Ni-CeO_2_ on HVOF coated NiCoCrAlY-modified aluminide	Vacuum treatment	[6]
Diffusion	Ni-based superalloy	Electroplated Pt-Zr composite-modified aluminide	Above pack	[44]
Diffusion	Ni-based superalloy	Electroplated Pt-Hf composite-modified aluminide	Gas-phase	[56]
Diffusion	Hastealloy X superalloy	Electroplated of Ni-modified aluminide	Slurry	[36]
		Electroplated Ni-50Co-modified aluminide	Slurry	
Diffusion	IN 792 Ni-based superalloy	Electroplated Pt-modified aluminide	Out of pack cementation	[21]
Diffusion	Ferritic-Martensitic	Electroplated Ni-modified aluminide	Packed cementation	[51]
Diffusion	second-generation SX Ni-based superalloy	Electroplated Pt-modified aluminide	Vacuum diffusion treatment	[57]
Diffusion	MAR M247 Ni superalloy	Electroplated (Pt + Pd)-modified aluminide	Low activity chemical vapor deposition aluminising	[50]
Diffusion	MAR M247 Ni superalloy	Electroplated Pd-Zr-doped modified aluminide	Low activity chemical vapor deposition aluminising	[50]
Diffusion	MAR M247 Nisuperalloy	Electroplated Pd-Hf-doped modified aluminide	Low activity chemical vapor deposition aluminising	[50]
Diffusion	MAR M247 Ni superalloy	Electroplated (Pt + Pd)-Zr-doped modified aluminide	Low activity chemical vapor deposition aluminsing	[50]
Diffusion	MAR M247 Ni superalloy	Electroplated (Pt + Pd)-Hf-doped modified aluminide	Low activity chemical vapor deposition aluminising	[50]
Overlay	NiCoCrAlY (As prepared)	Electroplated Pt on NiCoCrAlY	Vacuum Annealing at 1000 °C	[58]
	NiCoCrAlY (commercial)			
Overlay	Ni-based superalloy	Co-deposition of CrAlY-based particles in Ni, Co, Ni + Co metal matrix by electrodeposition	Diffusion heat treatment	[24]
Diffusion	Second generation Ni-based single crystal superalloy	Electrodeposited acidic Pt-modified aluminide	Gas-phase	[23]
		Electrodeposited basic Pt-modified aluminide		
Overlay	Ni-based single crystal superalloy	Electroplated Pt on arc ion plated NiCoCrAlY	Vacuum annealing at 1070 °C	[53]
		Arc ion plated NiCoCrAlY on electroplated Pt		
Overlay	Ni-based single crystal superalloy	Electroplated Pt on arc ion plated NiCoCrAlYSi	Vacuum annealing at 1070 °C	[7]
		Arc ion plated NiCoCrAlYSi on electroplated Pt		

## Data Availability

No new data were created or analyzed in this study.
